# Computational assessment of stomach tumor volume from multi-slice computerized tomography images in presence of type 2 cancer

**DOI:** 10.12688/f1000research.14491.2

**Published:** 2018-10-09

**Authors:** Gerardo Chacón, Johel E. Rodríguez, Valmore Bermúdez, Miguel Vera, Juan Diego Hernández, Sandra Vargas, Aldo Pardo, Carlos Lameda, Delia Madriz, Antonio J. Bravo

**Affiliations:** 1 Universidad Simón Bolívar, Facultad de Ingeniería, Cúcuta, 540004, Colombia; 2 Grupo de Automatización y Control, Universidad de Pamplona, Cúcuta, 540004, Colombia; 3 Universidad Simón Bolívar, Facultad de Ciencias Básicas y Biomédicas, Cúcuta, 540004, Colombia; 4 Grupo de Investigación en Procesamiento Computacional de Datos, Universidad de Los Andes-Táchira, San Cristóbal, 5001, Venezuela; 5 Universidad Nacional Experimental Politécnica Antonio José de Sucre, Barquisimeto, 3001, Venezuela; 6 Programa Calidad y Productividad Organizacional, Decanato de Investigación, Universidad Nacional Experimental del Táchira, San Cristóbal, 5001, Venezuela

**Keywords:** Stomach tumor, type 2 cancer, medical imaging, multi–slice computerized tomography, image enhancement, region growing method, marching cubes, three-dimensional reconstruction

## Abstract

**Background: **The multi–slice computerized tomography (MSCT) is a medical imaging modality that has been used to determine the size and location of the stomach cancer. Additionally, MSCT is considered the best modality for the staging of gastric cancer. One way to assess the type 2 cancer of stomach is by detecting the pathological structure with an image segmentation approach. The tumor segmentation of MSCT gastric cancer images enables the diagnosis of the disease condition, for a given patient, without using an invasive method as surgical intervention.

**Methods:** This approach consists of three stages. The initial stage, an image enhancement, consists of a method for correcting non homogeneities present in the background of MSCT images. Then, a segmentation stage using a clustering method allows to obtain the adenocarcinoma morphology. In the third stage, the pathology region is reconstructed and then visualized with a three–dimensional (3–D) computer graphics procedure based on marching cubes algorithm. In order to validate the segmentations, the Dice score is used as a metric function useful for comparing the segmentations obtained using the proposed method with respect to ground truth volumes traced by a clinician.

**Results:** A total of 8 datasets available for patients diagnosed, from the cancer data collection of the project, Cancer Genome Atlas Stomach Adenocarcinoma (TCGASTAD) is considered in this research. The volume of the type 2 stomach tumor is estimated from the 3–D shape computationally segmented from the each dataset. These 3–D shapes are computationally reconstructed and then used to assess the morphopathology macroscopic features of this cancer.

**Conclusions:** The segmentations obtained are useful for assessing qualitatively and quantitatively the stomach type 2 cancer. In addition, this type of segmentation allows the development of computational models that allow the planning of virtual surgical processes related to type 2 cancer.

## Introduction

Computed tomography (CT) is a 3-D medical imaging tool, with extensive beneficial impact on the diagnosis, characterization and explanation of complex health issues ^1^. The latest development in spiral CT with electrocardiogram gating technology is with multislice CT (MSCT) which has allowed for the acquisition of large volumes data and for the obtainment of dynamic volume imaging ^2, 3^.

MSCT of the abdomen has been used to determine the size and location of stomach cancers ^4^. This imaging modality has allowed for the staging of stomach cancer tumors. In general, MSCT is considered the best modality for the staging of gastric cancer and it allows for the assessment of local tumor extension, nodal disease and metastases through a non-invasive clinical procedure ^5^.

The ability of multi-detector systems to acquire wider areas of the abdomen than simple detector systems prevents the generation of image motion artifacts that are mainly due to the long breath-holds required. In this sense, the total abdomen acquisition requires shorter acquisition times, which decreases the amount of contrast product that needs to be used and increases the resolution of the 3-D images.

The manual assessment of the volume of adenocarcinoma of the stomach by CT requires the setting of the window width and window level in Hounsfield units, values that are generated by the subjective estimation of the clinician. In addition, the tumor must be delineated manually in each slice of the 3-D MSCT image. Following this, the area of the tumor is calculated according to the delineated region on each axial image plane. Finally, each area of the contiguous transverse tumor slice is summed to compute the whole-tumor volume.

To analyze the features and/or spatial distribution of functional regions of anatomic tissues or organs from medical images, segmentation, as an image processing technique, has been extensively used ^6^. Segmentation has been also used as preprocessing technique to extract the information required for processing techniques, such as diagnosis or quantification ^7^, visualization ^8^, and compression, storage and transmission ^9, 10^. The objective of segmentation is organizing and grouping the set of shapes contained in the images using the proximity, similarity and continuity of the shapes as the organization and grouping criteria ^11, 12^.

The two basic kinds of medical image segmentation techniques are based on the delineation of a curve that defines the anatomical structures ^13^, and the application of pattern classification methods ^14^. Both kinds allow for representation of the image as a non-overlapped set of two regions (the subject of interest and the background).

**Figure 1.**
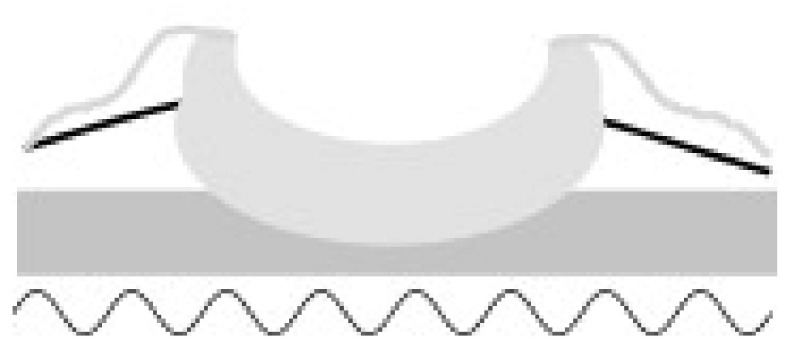
Type 2 cancer according Borrmann classification.

According to the Borrmann classification ^15^, type 2 cancer is an ulcerated but circumscribed advanced cancer. This cancer type is ulcerated, with partial marginal elevation and partial diffuse dissemination; it is frequently located in the antro and lesser curvature. Figure 1 shows the shape of type 2 cancer.

**Figure 2.**
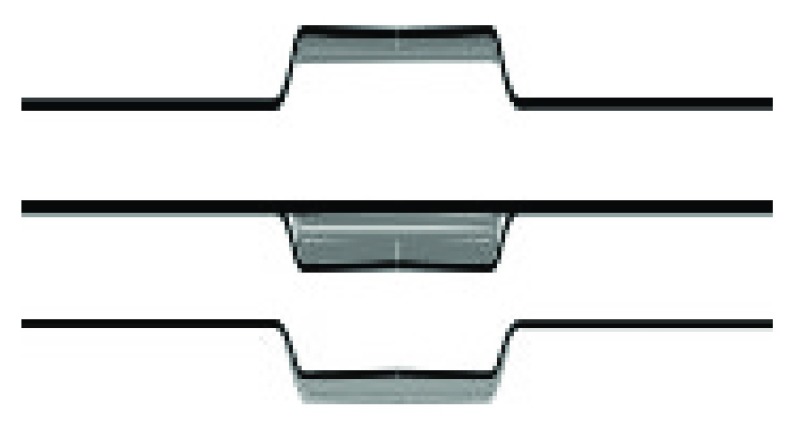
Japanese sub-classification of type 2 cancer.

The Japanese Gastric Cancer Association ^16, 17^ typified type 2 cancer as an advanced cancer that deepens and invades the muscular or subserosa layer. Type 2 or superficial cancer is classified as elevated or slightly elevated (less than 5 mm), flattened or flat, and depressed ( Figure 2).

This study presents the outcome of the development of a computational approach for assessing of type 2 stomach cancer from MSCT abdominal images. The approach consists of three stages. The initial stage, an image enhancement, consists of a method for correcting non-homogeneities present in the background of MSCT images. Next, a segmentation stage using a clustering method allows to obtain the adenocarcinoma morphology. In the third stage, the pathology region is reconstructed and then visualized with a 3-D computer graphics procedure based on marching cubes

The computational approach proposed herein adds to the academic contributions to the medical field, specifically the diagnosis and/or treatment of pathologies that require further scientific advances to increase the rate of curability. The social impact of this research is notable, because MSCT is a diagnostic technique with a lower cost than more invasive methods used to assess gastric cancer. This converges with the recommendations of the Pan American Health Organization ^18^, the World Health Organization ^19^ and the International Agency for Research on Cancer ^20^ for the prevention and control of this disease in order to facilitate the application of treatment methods based on scientific data.

The main objective of this research is to propose a computational approach to automatically detect the morphopatological shape of adenocarcinoma of the stomach. The proposal is based on a sequential design which involves image enhancement, segmentation, and three-dimensional image visualization. After the application of the proposed approach, a detailed analysis, both in a quantitative and a qualitative way is presented, by providing a measure of performance, and the assessment of several qualitative type 2 cancer features.

## Methods

The minimum system requirements needed to run the MatLab scripts contianed within this study are: 2.2 GB of HDD space for MATLAB only, 4–6 GB for a typical installation, any Intel or AMD x86 processor, 4 GB of memory RAM; no specific graphics card is required.

### Image source

The dataset considered in this research was obtained from The Cancer Genome Atlas–Stomach Adenocarcinoma (TCGA–STAD) ^21^. The dataset connects cancer phenotypes to genotypes using medical images matched with subjects from TCGA ^22, 23^. Table 1 shows the descriptive phenotypes and histological parameters of eight patients from TCGA–STAD with type 2 cancer. The TCGA-STAD dataset is composed of a single series, namely TCGA-STAD-VQ. The eight datasets used correspond with the patients of the series TCGA-STAD-VQ with type 2 cancer.

**Table 1.** Parameters of series TCGA–VQ- *XXXX*.

Case	Gender	Cancer location	T	N	M	Stage
A8DL	Female	Cardia/proximal	T3	N0	M0	IIA
A8E3	Male	Antro/distal	T3	N0	M0	IIA
A8P5	Male	Fundus/body	T3	N0	M0	IIA
A8P8	Female	Fundus/body	T4a	N0	M0	IIB
A8PB	Female	Antro/distal	T3	N0	M0	II
A94U	Male	Antro/distal	T4a	N0	M0	IIB
AA6F	Male	Cardia/proximal	T3	N1	M0	IIB
AA6G	Male	Cardia/proximal	T3	N0	M0	IIA

T, tumor stage; N, lymph node stage; M, metastasis stage.

### Phase 1: Image enhancement

The software used in this phase was developed within the framework of the present investigation and corresponds with a MatLab script (MatLab R2012a). This script corresponds with Enhancement Software, and is available on Zenodo ^24^.

As the gastric mucosa has many folds and is formed by connective tissue that joins the muscle and the mucosa, the abdominal tomography images produced in the interface between the mucous and the contrast agent are, in certain regions, not homogeneous, and consequently the tumor is shown with unclearly differentiated edges.

An enhancement approach is required to improve the adenocarcinoma information with respect to the non-homogeneous background. To correct the non-homogeneities and to enhance the adenocarcinoma, the look-up table (LUT)-based method ^25^ is used. This method has been used to improve confocal microscopy ^26^ and X-ray rotational angiography ^27^ images. The LUT is constructed according to the following procedure:

1. Choose *n* non-homogeneous images.
2. Determine the frequency percentage vector *f ^j^* = [ *f ^j^*_0_ *f ^j^*_1024_ *f ^j^*_2048_ *f ^j^*_3072_ *f ^j^*_4095_] (1 ≤ *j* ≤ *n*) for each image in the histogram. These frequencies are associated with the gray level vector, **Level** = [ *Level*_0_ *Level*_1_ *Level*_2_ *Level*_3_ *Level*_4_] = [0 1024 2048 3072 4095].
3. Obtain the average for the frequency percentage vector for all images, $F_{Level}=\;F_{Level_{k}}\;\sum_{j=1}^{n}f_{Level_{k}}^{j},\forall \;0\leq k\leq 4$, k is a positive integer.
4. Construct a LUT as a transfer function defined by the concatenation of four linear transformations. Each linear function is constructed using $Input_{LUT}^{k}=Level_{k},\forall \;0\leq k\leq 4$ as input and $Output_{LUT}^{k}=\sum_{l=0}^{k}F_{Level_{k}},\forall \;0\leq k\leq 4$ as input and output, respectively.

**Figure 3.**
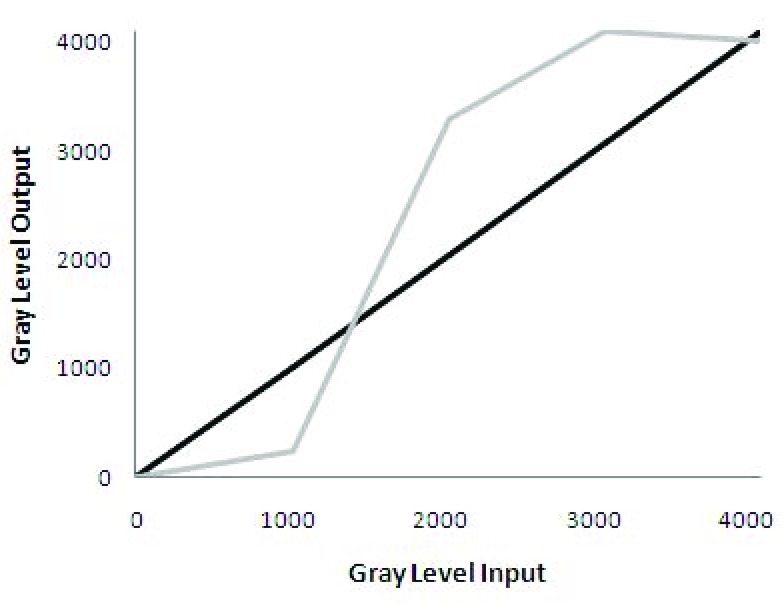
Linear look-up table.

Figure 3 shows both LUTs, the original (black) and the constructed using the previous procedure (gray).

Once corrected, the image is smoothed using a Gaussian filter with a spread factor σ. This parameter is set to the standard deviation value of the corrected image. The relationship between corrected and smoothed images is obtained using a simple linear regression model ^28^.

### Phase 2: Segmentation

Segmentation was performed using VolView 3.4 for Linux, 64 bit.

The segmentation is based on a simple linkage region growing technique. The following procedure is used:

1. A voxel is tagged as a seed voxel of a new cluster when its intensity value is lower than the standard deviation of the enhanced image, and it is still unlabeled (the voxel is not associated to any cluster). The procedure is ended when all voxels are labeled.
2. All neighbor voxels of the new cluster are eligible for merging. For each neighbor voxel, every voxel in an 8-neighborhood is considered. The neighbor voxel is joined to the current cluster if i) the neighbor voxel is still unlabeled; and ii) the intervoxel distance of the edge voxel and the neighbor voxel is below the standard deviation of the enhanced image. When the current cluster stops growing, go back to point 1.

The region growing algorithm of the multi-platform application VolView considers two parameters, namely, the neighborhood size for the region growing ( *l*) and scale factor of grouping (τ).The clustering algorithm is applied by varying the value of these parameters in order to tune them.

This procedure is applied to the enhanced image to obtain two regions, the tumor and the background.

### Phase 3: Reconstruction of tumor surface

After the segmentation process, the reconstruction of the pathological surface is performed using the Visualization Toolkit (VTK-6.3.0) ^29^. VTK is an open-source library available for image processing and 3-D scientific visualization used by many researchers and worldwide developers. The reconstruction algorithm is designed according to an object-oriented computational model, which is developed with the C-class library contained in VTK ^30^. This reconstruction procedure only requires the segmented volume as input parameter.

The tumor wall is reconstructed using the marching cubes algorithm ^31^. Marching cubes have long been used as a standard indirect volume-rendering approach to extract iso-surfaces from 3-D volumetric data. The algorithm was developed by Lorensen and Cline ^32^ and has this name because it takes eight neighboring locations simultaneously to constructing an imaginary cube, generating the necessary polygons to reconstruct the surface.

### Validation of the segmentation

The validation of the proposed segmentation technique is performed by quantifying the difference between the estimated pathological shape with respect to a ground truth shape, traced by an expert (V.B.). The difference is estimated using the Dice coefficient, which quantifies the degree of overlap between two volumes ^33^. Another MatLab script (in MatLab version R2012a) was also written within the framework of the present investigation to validate the proposed segmentation approach. Version 1.0 of this script is available on Zenodo ^34^; its input parameters correspond with the type 2 cancer estimated shape and the ground truth shape.

## Results

The results obtained from this research are in part based upon data generated by TCGA Research Network: http://cancergenome.nih.gov/.

**Figure 4.**
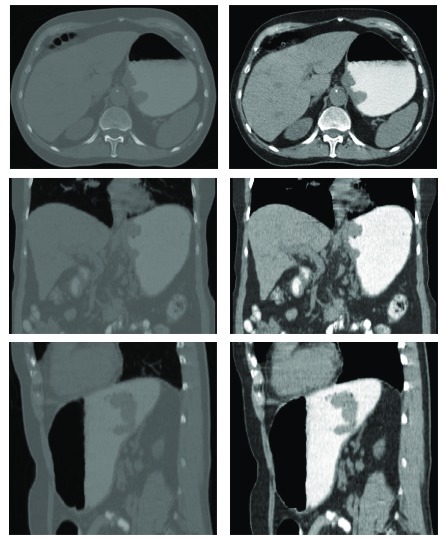
TCGA–VQ–A8DL dataset. First column, original images; second column, corrected images.

The MSCT 3-D image of the TCGA–VQ–A8DL dataset is used to illustrate the proposed three-phase approach. The non-homogeneous background correction procedure described in *Phase 1: Image enhancement* is applied to the dataset ( Figure 4). The background appears to be more homogeneous, whereas the associated type 2 cancer tumor information is enhanced.

The parameter *n* required for the procedure of images enhancement ( *Phase 1: Image enhancement*), which ** corresponds to the amount non-homogeneous images, is chosen as 20% of the slices of each tomographic volume to be analyzed. The TCGA–VQ–A8DL dataset contains an MSCT volume of 512×512×101; since the volume has 101 slices, *n* corresponds to 20 of those slices.

The seed voxel is used to start the region-growing segmentation process. This seed is established in the bi-dimensional image (MSCT slice). A manual process performed by a clinician is applied to locate the volume slice, where the adenocarcinoma area is visually maximized.

In the parameters tuning procedure, for τ, all the values included in the interval [0 10] with a step size of 0.1 are evaluated, meanwhile *l* varies between 1 and 20 with step size of 1. For each set of parameters, the resulting segmented structures are compared with the corresponding structures traced by a clinician. The differences are estimated using the Dice coefficient. The parameters value that maximized the Dice coefficient are chosen for the proposed segmentation method. These same parameters values are considered to segment others dataset.

**Figure 5.**
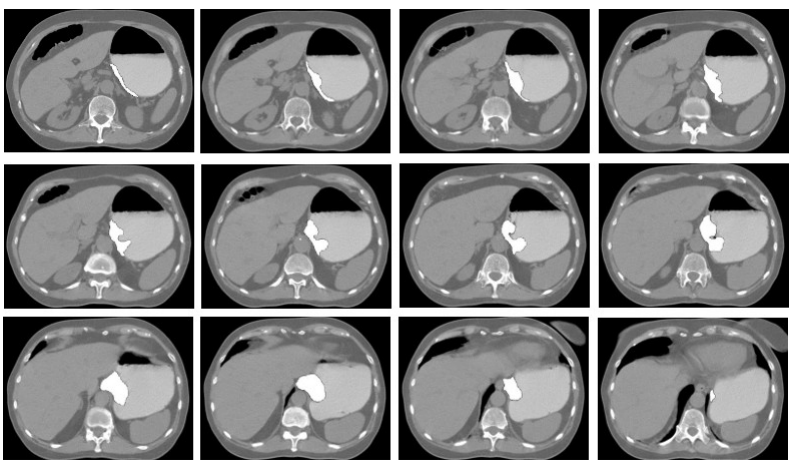
Adenocarcinoma area detection for TCGA–VQ–A8DL dataset.

Figure 5 shows the results of this method applied to the dataset TCGA–VQ–A8DL. In each row, four MSCT slices (the axial anatomical view) are shown in the image volume. The adenocarcinoma contour is indicated by a black dash-dotted line. Regions extracted using the proposed segmentation approach are indicated by white areas.

**Figure 6.**
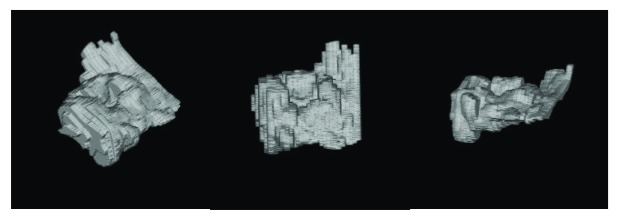
3-D reconstruction of adenocarcinoma shape of the TCGA–VQ–A8DL dataset.

Figure 6 shows the 3-D reconstruction obtained using the procedure based on the marching cubes algorithm. The input of the reconstruction algorithm is the adenocarcinoma shape, segmented using the procedure based on a region-growing technique. Table 2 shows the volumes quantified from the reconstructions.

**Table 2.** Adenocarcinoma volumes.

Case	Volume, mm ^3^
A8DL	52.92×10 ^3^
A8E3	37.12×10 ^3^
A8P5	24.37×10 ^3^
A8P8	61.90×10 ^3^
A8PB	47.43×10 ^3^
A94U	23.31×10 ^3^
AA6F	10.37×10 ^3^
AA6G	36.28×10 ^3^

The comparison between the segmented structures and the cancer shapes delineated by the clinician is performed based on the Dice coefficient. The Dice coefficient obtained (mean +/- standard deviation) for the eight datasets of the 3-D images is 91.54% +/- 5.26%, with a maximum value of 97.31% and a minimum value of 83.47%. Accordingly, the maximum estimated error of segmentations corresponds with 16.53%, and the minimum estimated error with 2.69%

## Discussion

The method proposed in this study can be used to generate 3-D segmentation of stomach adenocarcinoma. These segmentations are useful for monitoring the application of cancer treatment methods based on scientific data, since they allow for the calculation of certain clinical quantitative descriptors, such as volume, and for the assessment of several qualitative type 2 cancer features.

The method is tested on eight gastric cancer datasets. The estimated error for all MSCT images is reported. The proposed application for detecting type 2 cancer tumors generates the highest Dice score values.

The calculated volume represents a parameter that is more representative of the real size of all the segmented pathological structure as the descriptor is calculated from a realistic 3-D computational model. This is particularly true as tumor volume is an important indicator of lymph node metastasis in advanced gastric cancer. From Figure 6, several macroscopic features associated with type 2 cancer can be validated, such as: circumscribed, with well-defined borders, and ulcerated.

The segmentations generated by the proposed method can be useful in various scenarios such as:

1. Academic–didactic: Promoting, deepening and potentiating the study of the real pathology.
2. Research: Design and development of robust, automatic and efficient segmentation methods.
3. Clinical: Supporting the planning of therapeutic and surgical processes associated with stomach cancer.

## Conclusions

A three-phase approach has been developed based on an image enhancement and region-growing clustering technique for segmenting stomach tumors associated with type 2 cancer. The segmentations obtained are useful for assessing this pathology. In addition, this type of segmentation allows for the development of computational models that allow the planning of virtual surgical processes related to type 2 cancer.

A region-growing clustering technique is controlled by a seed point located in a volume slice, which is propagated to the rest of slices to segment the entire MSCT volumes. The validation of the obtained segmentations shows that the pathological representation obtained using the proposed method exhibits the highest correlation to the type 2 cancer shape traced by a clinician.

In future work, a more complete validation is necessary, considering more large-image datasets, and including a comparison of estimated parameters describing the adenocarcinoma volume with respect to results obtained using other measurement techniques.

## Data availability

The TCGA-STAD type 2 stomach cancer dataset series is available from: https://portal.gdc.cancer.gov/projects/TCGA-STAD, using the parameters TCGA-VQ- *XXXX*. 

## Software availability

**Enhancement software available from/archived source code at time of publication:** https://doi.org/10.5281/zenodo.1253039 ^24^.

**MatLab script used to compute the Dice coefficient available from/archived source code at time of publication:** https://doi.org/10.5281/zenodo.1289908 ^34^.

**License:** Creative Commons Attribution-ShareAlike 4.0 International.
