# Supplementary material for: Computational assessment of stomach tumor volume from multi-slice computerized tomography images in presence of type 2 cancer
Source: F1000Res. 2018 Oct 9;7:1098. Originally published 2018 Jul 17. [Version 2] doi: 10.12688/f1000research.14491.2 (PMC6234734; doi:10.12688/f1000research.14491.2)
Supplement: Peer review history

# Peer review: Referee response for version 2

**DOI:** 10.5256/f1000research.18013.r40121

**Published:** 2018-11-07

**Authors:** Pablo Guillen-Rondon

**Affiliations:** 1 Center for Advanced Computing and Data Science, University of Houston, Houston, TX, USA

The authors develop a computational approach to assess the volume of the stomach tumor. The tumor volume is assessed from multi-slice computerized tomography images of patients with type 2 cancer.

 First, the approach automatically detects the morphopatological shape of adenocarcinoma of the stomach. In this sense, an image enhancement technique to correct non homogeneities in the image's background is initially considered. After, a regions growing based technique is used to segment the pathology structure. Then, the segmented structure is reconstructed using a marching cubes based algorithm implemented with Toolkit VTK-6.3.0. The proposed method is well explained. 

 The validation of the proposed approach is performed based on Dice coefficient. The segmented structures are compared with respect to the cancer shapes delineated by the clinician by means this coefficient. From this comparison the authors reported that their computational approach generated high Dice score values, and therefore, low error values.

I have read this submission. I believe that I have an appropriate level of expertise to confirm that it is of an acceptable scientific standard.

# Peer review: Referee response for version 1

**DOI:** 10.5256/f1000research.15776.r37689

**Published:** 2018-09-28

**Authors:** Fred Prior

**Affiliations:** 1 Department of Biomedical Informatics, University of Arkansas for Medical Sciences (UAMS), Little Rock, AR, USA

The data used in this study is available by not from the source noted in the paper. The data is available from the Cancer Imaging Archive (https://wiki.cancerimagingarchive.net/display/Public/TCGA-STAD) not from the GDC portal as described.

 The results section states that DICE coefficients are used for comparison of the machine segmented lesions and human segmented lesions which serve as a model of truth. However the results are reported as: "The error obtained (mean +/- standard deviation) for the eight datasets of the 3-D images is 91.54% +/- 5.26%, with a maximum value of 97.31% and a minimum value of 83.47%. I believe these may be the calculated DICE coefficients and not "error" estimates. Errors between the two segmented lesions this large would indicate complete failure of the technique.

 In general the techniques proposed are not novel but rather application of well known and proven techniques to a small data set.

I have read this submission. I believe that I have an appropriate level of expertise to state that I do not consider it to be of an acceptable scientific standard, for reasons outlined above.

# Response

**Published:** 2018-09-28

**Authors:** Gerardo Chacon

**Affiliations:** Universidad Simon Bolivar Colombia, Colombia

The authors would like to thank to Dr Fred Prior for his comments about our paper.

 The hyperlink to the data source noted in the Version 1 of our paper, is modified to

 https://wiki.cancerimagingarchive.net/display/Public/TCGA-STAD (Cancer Imaging Archive).

 In the section **Results,** the last paragraph is modified according to the following text:

 The comparison between the segmented structures and the cancer shapes delineated by the clinician is performed based on the Dice coefficient. The Dice coefficient obtained (mean +/- standard deviation) for the eight datasets of the 3-D images is 91.54% +/- 5.26%, with a maximum value of 97.31% and a minimum value of 83.47%. Accordingly, the maximum estimated error of segmentations corresponds with 16.53%, and the minimum estimated error with 2.69%.

 Additionally, in the section **Conclusions**, the last paragraph is modified as follows:

 In future work, a more complete validation is necessary, considering a more-large image datasets, and including a comparison of estimated parameters describing the adenocarcinoma volume with respect to results obtained using other measurement techniques.

 As a final remark, we would like to comment, that the proposed methods for enhancement and segmentation the CT gastric images are conceptually simple and require minimal user interaction, which allows decreasing the intersubjective probability or personal uncertainty introduced by the methods users. And, as verified with the help of the comments of another of the referees the enhancement algorithm is the core of the paper. This algorithm allows to control the non-homogeneities of the abdominal tomography images.produced in the interface between the mucous and the contrast agent and consequently, the technique allows to improve the information associated with the unclearly differentiated edges of the tumor.This improvement is achieved by a scheme based on a lookup table. This framework focuses on analyzing the average information of a sample images and then the optimal transfer function useful to diminish the impact of non-homogeneities in the segmentation is constructed. Hence, that we consider our method, conceptually simple and of low computational cost. In this sense, an interesting image processing technique is proposed. And finally, the application of our method to more-large image data sets rather than a research process corresponds to the putting process into practice of our method. 

 The Authors.

# Peer review: Referee response for version 1

**DOI:** 10.5256/f1000research.15776.r36172

**Published:** 2018-08-17

**Authors:** Jorge Brieva

**Affiliations:** 1 Faculty of Engineering, Panamerican University, Mexico City, Mexico

Authors propose a method to asses a stomach tumor volume.  They use multi-slice computerized tomography images that present a cancer of type 2. 

 The method include three steps: first, an image enhancement to correct non homogenities  in the background.The enhancement is based on the LUT. Then, the segmentation is carried out by the region growing technique widelly used in segmentation problems. Finally, a reconstruction is made using the Toolkit VTK-6.3.0.

 The validation use the Dice coefficient to compare to the clinical manual segmentation. 

 The method is well explained, however more detail would  be suitable to the enhancement algorithm that seems the core of the paper. In paticular, in the Equation of Flevel the index j is well used afeter the sum? More details would be benefics to understand the method. 

 In the segmentation method there are some parameters to tune?  If it is the case it is suitable to add in the text. 

I have read this submission. I believe that I have an appropriate level of expertise to confirm that it is of an acceptable scientific standard.

# Response

**Published:** 2018-09-28

**Authors:** Gerardo Chacon

**Affiliations:** Universidad Simon Bolivar Colombia, Colombia

The authors would like to thank to Dr Jorge Brieva for his valuable comments which helped to improve the manuscript.

 In order to explain in more detail the enhancement algorithm, this algorithm constitutes the core of our paper, the procedure for constructing the LUT described in the Version 1 of our paper is modificated as follow

 1. Choose n non-homogeneous images.

 2. Determine the frequency percentage vector f^j = [f^j_0 f^j_1024 f^j_2048 f^j_3072 f^j_4095] (1<= j <= n) for each image in the histogram. These frequencies are associated with the gray level vector, Level = [Level_0 Level_1 Level_2 Level_3 Level_4] = [0 1024 2048 3072 4095].

 3. Obtain the average for the frequency percentage vector for all images, F_Level = F_(Level_k) =Sum_(j=1)^n f_Level_k^j, forall 0<= k <= 4.

 4. Construct a LUT as a transfer function defined by the concatenation of four linear transformations. Each linear function is constructed using Input_LUT^k =Level_k, forall 0 <= k <= 4  and Output_LUT^k = Sum_(l=0)^k F_(Level_k ), forall 0 <= k <= 4, as input and output, respectively.

 On the other hand, in the section: **Phase 2: Segmentation**, the following information about the segmentation phase is added:

 The region growing algorithm of the multi-platform application VolView considers two parameters, namely, the neighborhood size for the region growing (l) and scale factor of grouping (tau). The clustering algorithm is applied by varying the value of these parameters in order to tune them. 

 Additionally, in the section **Results**, the following text is added:

 In the parameters tunning procedure, for tau, all the values included in the interval [0 10] with a step size of 0.1 are evaluated, meanwhile l varies between 1 and 20 with step size of 1. For each set of parameters the resulting segmented structures are compared with the corresponding structures traced by a clinicians. The differences are estimated using the Dice coefficient. The parameters value that maximized the Dice coefficient are chosen for the proposed segmentation method.These same parameters values are considered to segment others dataset.
